# Induction of Poly(ADP-ribose) Polymerase in Mouse Bone Marrow Stromal Cells Exposed to 900 MHz Radiofrequency Fields: Preliminary Observations

**DOI:** 10.1155/2016/4918691

**Published:** 2016-04-14

**Authors:** Qina He, Yulong Sun, Lin Zong, Jian Tong, Yi Cao

**Affiliations:** School of Public Health, Medical College of Soochow University, Suzhou, Jiangsu 215123, China

## Abstract

*Background*. Several investigators have reported increased levels of poly(ADP-ribose) polymerase-1 (PARP-1), a nuclear enzyme which plays an important role in the repair of damaged DNA, in cells exposed to extremely low dose ionizing radiation which does not cause measurable DNA damage.* Objective*. To examine whether exposure of the cells to nonionizing radiofrequency fields (RF) is capable of increasing messenger RNA of PARP-1 and its protein levels in mouse bone marrow stromal cells (BMSCs).* Methods*. BMSCs were exposed to 900 MHz RF at 120 *μ*W/cm^2^ power intensity for 3 hours/day for 5 days. PARP-1 mRNA and its protein levels were examined at 0, 0.5, 1, 2, 4, 6, 8, and 10 hours after exposure using RT-PCR and Western blot analyses. Sham-exposed (SH) cells and those exposed to ionizing radiation were used as unexposed and positive control cells.* Results*. BMSCs exposed to RF showed significantly increased expression of PARP-1 mRNA and its protein levels after exposure to RF while such changes were not observed in SH-exposed cells.* Conclusion*. Nonionizing RF exposure is capable of inducing PARP-1.

## 1. Introduction

Damage to the genetic material (DNA), due to normal endogenous metabolic processes, occurs in cells at a rate of up to 1,000,000 molecular lesions per cell per day [1]. Genotoxic agents are known to potentiate these lesions which include alterations in bases and single- and double-strand breaks (SSB-DSB) resulting in structural damage that can alter or eliminate the ability of the cells to transcribe the gene that the affected DNA encodes. In order to deal with problems under which the DNA is vulnerable to injury, an elaborate and complex set of surveillance mechanisms were evolved in eukaryotic cells to reverse and/or remove potentially deleterious damage. These include a cascade of signal transduction processes which consist of multiple interconnected pathways that transmit the damage signals and trigger responses to repair the DNA, cell cycle arrest, and apoptosis [2]. There is ample evidence that poly(ADP-ribose) polymerase (PARP), a family of nuclear enzymes in eukaryotic cells, plays an important role in genomic stability by regulating DNA repair, gene transcription, cell cycle progression, chromatin function, and cell death. Among these nuclear enzymes, PARP-1 is more abundant and acts as a “molecular nick sensor” to signal the cells about strand breaks in the DNA and to assist in their repair [3–11].

Numerous investigators have demonstrated that extremely low doses of ionizing radiation (IR) exposure in animal and human cells, in the absence of measurable induction of DNA damage, were able to alleviate the DNA damage induced by subsequent exposure to a high dose of IR or other similar genotoxic agents suggesting that efficient DNA repair mechanism(s) might be playing a role in such cells [12]. The evidence for one such mechanism was provided by the significantly increased PARP-1 mRNA expression and its protein levels in mice and cultured mouse lymphoma cells exposed to a nongenotoxic dose of IR, and such increase was negated when the mice were injected and the cells were treated with 3-aminobenzamide (3-AB), a potent inhibitor of PARP-1 [13, 14].

To the best of our knowledge, there were no published reports about whether nonionizing radiofrequency fields (RF) exposure is capable of inducing PARP-1 in mammalian cells. Nonetheless, the results from our more recent studies indicated that the whole body of mice and cultured mouse bone marrow stromal cells (BMSCs) exposed to 900 MHz RF for several days showed significantly reduced levels of strand breaks in the DNA as well as faster kinetics of their repair when challenged with genotoxic dose of *γ*-radiation or bleomycin (BLM), a radiomimetic chemotherapeutic drug [15]. Therefore, we have conducted a preliminary investigation on BMSCs to examine whether 900 MHz RF (continuous wave) exposure at 120 *μ*W/cm^2^ power intensity for 3 hours/day for 5 days is capable of inducing PARP-1. The rationale for using this power intensity was our earlier observation of significant survival advantage of lethally irradiated mice which were preexposed to 900 MHz RF at 120 *μ*W/cm^2^ power intensity compared to those which were preexposed to RF at 12 *μ*W/cm^2^ or 1200 *μ*W/cm^2^ [16]. The results obtained in this study were compared with those in sham-exposed (SH) control cells as well as in those exposed to 1.5 Gy *γ*-irradiation (GR, positive controls) and the data discussed.

## 2. Materials and Methods

The experimental protocol was approved by the institutional animal care and ethics committee of Soochow University.

### 2.1. Bone Marrow Stromal Cells (BMSCs)

In the current* in vitro* study, cultured BMSCs were used. BMSCs exhibit multiple characteristics/traits of a stem cell population including regulation of cytokine production and release of growth factors required for hematopoiesis which are considered a model of hematopoiesis [17]. The collection of bone marrow cells and the culture of stromal cells were described in detail in our earlier study [18]. Briefly, 4 adult Kunming mice were initially purchased from the Animal Center in Soochow University (Suzhou, Jiangsu, China; the Animal Care/Use Ethical Committee of Soochow University, Suzhou, China, has reviewed and approved our handling of animals). After 7 days of quarantine, the animals were sacrificed by cervical dislocation. From each mouse, bone marrow was flushed with phosphate-buffered saline (PBS, Gibco, Shanghai, China) and single cell suspension was prepared in complete IMDM medium (Iscove's modified Dulbecco's medium, Hyclone, Suzhou, China) containing 10% fetal bovine serum (FBS, Gibco, Shanghai, China), 100 units/mL penicillin, and 100 *μ*g/mL streptomycin (Bio Basic, Hangzhou, China). For each mouse, aliquots of approximately 2 × 10^5^ cells in 3 mL medium were placed in 30 mm Petri dishes (Nunc, Shanghai, China) and cultured for 48 h in an incubator (Heal Force Bio-Meditech, Hong Kong, China) at a temperature maintained at 37 ± 0.5°C with humidified atmosphere of 5% carbon dioxide and 95% air. Then, for each mouse, the nonadherent cells were discarded and the adherent BMSCs were cultured further in fresh complete medium. Cultured BMSCs in 3–6 passages, from a single mouse, were used in 3 independent investigations.

On the day before starting the RF and SH exposures, aliquots of approximately 5 × 10^5^ BMSCs/mL (8 mL total) were seeded into 24 separate 100 mm Petri dishes and were left in the incubator at a temperature maintained at 37 ± 0.5°C with humidified atmosphere of 95% air and 5% carbon dioxide. On the next day, the medium was replaced in all Petri dishes; 16 petri dishes were used for RF and SH exposure (8 Petri dishes each) for 3 hours/day for 5 days while the other 8 were left in the incubator for an acute exposure of GR (at the end of RF and SH exposures). The medium in all dishes was changed once during this time and the cells in all Petri dishes were confluent by 5 days.

### 2.2. RF and SH Exposures

The exposure system was built in-house at Soochow University, Suzhou (Jiangsu, China), and described in detail earlier [16]. Briefly, it consists of GTEM chamber (Gigahertz Transverse Electromagnetic Chamber; 5.67 m length, 2.83 m width, and 2.07 m height), a signal generator (SN2130J6030, PMM, Cisano sul Neva, Italy), and a power amplifier (SN1020, HD Communication, Ronkonkoma, NY). The continuous wave 900 MHz RF signal was generated, amplified, and fed into the GTEM chamber through an antenna (Southeast University, Nanjing, Jiangsu, China). The RF field inside the GTEM was probed using a field strength meter (PMM, Cisano sul Neva, Italy) to determine the precise position which provided the required 120 *μ*W/cm^2^ power intensity. The power was monitored continuously and recorded every 5 min in a computer controlled data logging system which indicated 12.178 ± 0.003 *μ*W/cm^2^ during the 3-hour RF exposure. The GTEM was installed in a room in which temperature was maintained at 37 ± 0.5°C (87% relative humidity, without CO_2_) and the temperature inside the GTEM was also similar during exposure of the cells to RF.

For RF exposure, the BMSCs in 8 separate Petri dishes (arranged in two rows of 4 each and all dishes touching each other) were placed on a nonconductive table/platform at a height of 100 cm at the precise location where the required power intensity of 120 *μ*W/cm^2^ was measured. The distance between Petri dishes and the exposure unit (probe) was 18 cm. At the input 120 *μ*W/cm^2^ power intensity and the direction of propagation of the incident field parallel to the plane of the medium, the peak and average SARs estimated were extremely low: they were 4.1 × 10^−4^ and 2.5 × 10^−4^ W/kg, respectively [19]. The RF exposure was 3 hours/day for 5 days. BMSCs in the other 8 separate Petri dishes were exposed in the GTEM chamber, without RF transmission, for 3 hours/day for 5 days, and these cells were used as SH-exposed control cells.

### 2.3. Gamma Radiation (GR)

The BMSCs in 8 Petri dishes (which were left in the incubator) were exposed to an acute dose of 1.5 Gy *γ*-radiation (Nordion, Ottawa, ON, Canada; dose rate: 0.5 Gy/min) from ^60^Co source which was located in another building. There was an interval of ~10 minutes between irradiation of the cells and their transport to the laboratory.

### 2.4. RT-PCR (mRNA Expression of PARP-1)

Immediately after RF and SH exposures (~10 minutes after GR exposure), the cells in all Petri dishes were kept in the incubator at a temperature maintained at 37 ± 0.5°C, with humidified atmosphere of 95% air and 5% carbon dioxide. At different intervals, namely, 0, 0.5, 1, 2, 4, 6, 8, and 10 hours, the cells in separate dishes were collected, washed in phosphate-buffered saline (PBS, Gibco, Shanghai, China), and divided into 2 aliquots. The cells in one aliquot were utilized to extract total RNA using Trizol agent (Tiangen Biotech, Beijing, China) while those in the other aliquot were used for protein extraction (see below). The cDNA was synthesized from the messenger RNA (mRNA) using the Thermo Scientific RevertAid First Strand cDNA Synthesis Kit (Thermo Fisher Scientific, Waltham, MA, USA) according to the manufacturer's instructions. This was followed by RT-PCR amplification with an initial step of 2 minutes at 50°C and 10 minutes at 95°C, followed by 40 cycles of 15 s at 95°C and 1 min at 60°C (ABI Prism 7500 Sequence Detection System, Applied Biosystems, USA).

The primers used for PARP-1 were the following:

 Forward: 5′-CCATCGACGTCAACTACGAG-3′.
 Reverse: 5′-GTGCGTGGTAGCATGAGTGT-3′.

The primers for glyceraldehyde-3-phosphate dehydrogenase (GAPDH, Good Science, Shanghai, China), a housekeeping gene, were also included as controls:

 Forward: 5′-CATGGCCTTCCGTGTTCCTA-3′.
 Reverse: 5′-CCTGCTTCACCACCTTCTTGAT-3′.

The PCR products were stained with Fast Start Universal SYBR Green Master (Roche Group, Basel, Switzerland) as double-stranded DNA-specific fluorescent dye. PARP-1 expression was normalized by subtracting the mean of GAPDH Ct value from RF-, SH-, and GR-Ct value (ΔCt). The fold change value was calculated using the expression 2^−ΔΔCt^, where ΔΔCt represents ΔCt_treatment  group_ − ΔCt_control  group_. The results represented were average (± standard deviation) from three independent experiments.

### 2.5. Western Blot Analysis (PARP-1 Protein)

Protein extracts were prepared (from the cells in the second aliquot) by lysing the cells in lysis buffer containing 50 mM Tris (pH 7.4), 150 mM sodium chloride, 1% Triton X-100, 1% sodium deoxycholate, 0.1% sodium dodecyl sulfate, and 1 mM phenylmethylsulfonyl fluoride (all obtained from Beyotime, Shanghai, China). The cell lysates were centrifuged at 14,000 ×g for 5 minutes at 4°C and the supernatant containing solubilized proteins was collected. The protein concentration in all samples was determined by the BCA protein assay kit (Beyotime, Shanghai, China). Equal amounts of proteins (40 *μ*g per lane) were loaded, separated by 8% sodium dodecyl sulfate-polyacrylamide gels (SDS-PAGE), and then transferred to polyvinylidene difluoride (PVDF) membranes (Millipore Corporation, Billerica, MA, USA). The membranes were blocked for 2 hours in 5% fat-free dry milk (Yili Industrial Group, Inner Mongolia, China) containing Tween 20-Tris-buffered saline (TTBS). After blocking, the membranes were incubated with primary antibodies, namely, rabbit monoclonal anti-PARP-1 (Cell Signaling, Boston, MA, USA) and mouse monoclonal anti-GAPDH (Good Science, Shanghai, China), overnight at 4°C and washed three times in TTBS. The membranes were further incubated with horseradish peroxidase-conjugated antibodies for PARP-1 and GAPDH (Beyotime, Shanghai, China) for 1.5 hours at room temperature. This was followed by washing the membranes three times with TTBS. The immunoreactive proteins on the membranes were detected with enhanced chemiluminescence reagents (Millipore Corporation) using G:BOX Chemi XRQ (Syngene, UK). The blots were quantified by densitometry and normalized for GAPDH to correct for differences in loading of the proteins in RF-, SH-, and GR-exposed cells. The results presented were average (± standard deviation) from three independent experiments.

### 2.6. Statistical Analysis

The results were subjected to statistical analyses of variance (ANOVA) test using Statistical Product and Service Solutions for Windows [20]. Comparisons were made between cells exposed to RF and SH, RF, and GR and a *p* value of <0.05 was considered as significant difference between the 2 groups.

## 3. Results

**Figure 1:**
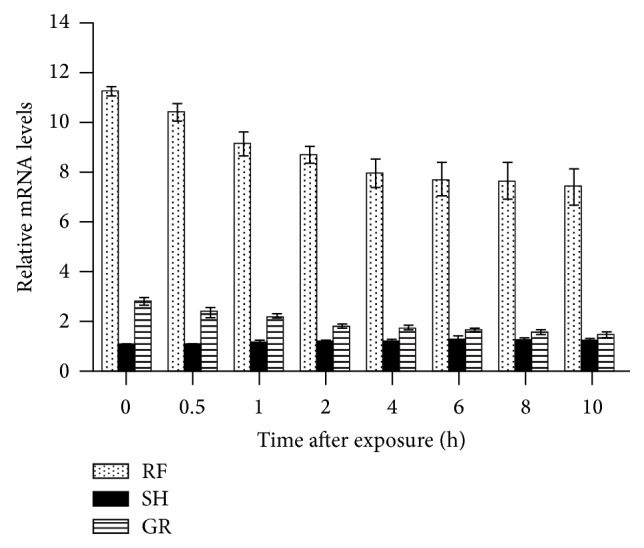
Expression levels of poly(ADP-ribose) polymerase-1 (PARP-1) mRNA in bone marrow stromal cells following exposure to 900 MHz radiofrequency fields (RF), sham (SH), and 1.5 Gy gamma radiation (GR).

The PARP-1 mRNA expression profiles, ascertained from RT-PCR analyses, in BMSCs exposed to RF, SH, and GR are presented in Figure 1 and Table 1. The average coefficients of variability (CV) in PARP protein levels in RF-, SH-, and GR-exposed cells were 6.2% (range: 1.8–9.7%), 7.3% (range: 0.0–11.9%), and 5.4% (range: 3.9–8.6%), respectively (CV was taken into consideration while calculating the significant difference between groups, *p* values). The data indicated that the expression levels were significantly higher/upregulated in RF-exposed cells at 0 hours compared with that in SH- and GR-exposed cells, and this was sustained at 0.5, 1, 2, 4, 6, 8, and 10 hours after exposure. However, the levels decreased slowly over time but were significantly higher even at 10 hours after RF exposure compared to those in SH-exposed cells. Compared with SH-exposed cells, those exposed to GR had significantly higher mRNA expression levels at 0 (i.e., ~10 minutes after exposure), 0.5, 2, and 4 hours but decreased over time at 6, 8, and 10 hours after exposure where the difference between the two groups of cells was not significantly different.

**Figure 2:**
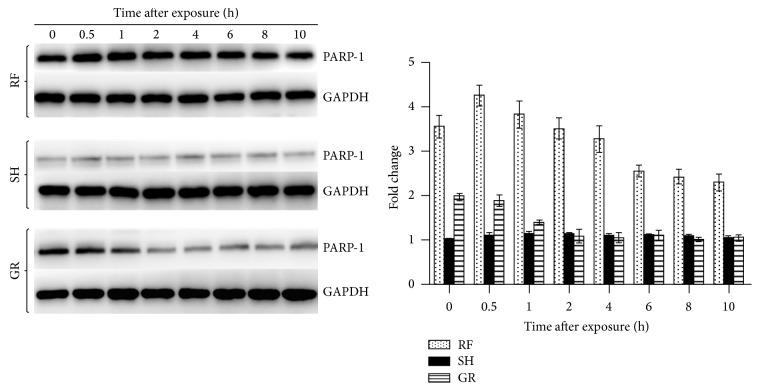
Poly(ADP-ribose) polymerase-1 (PARP-1) protein levels in bone marrow stromal cells following exposure to 900 MHz radiofrequency fields (RF), sham (SH), and 1.5 Gy gamma radiation (GR).

The levels of PARP-1 protein, assessed from Western blot analysis, at all times examined, were presented in Figure 2 and Table 2. The average coefficients of variability (CV) in PARP protein levels in RF-, SH-, and GR-exposed cells were 7.1% (range: 5.3–8.9%), 3.5% (range: 0.0–6.9%), and 7.8% (range: 3.3–14.8%), respectively (CV was taken into consideration while calculating the significant difference between groups, *p* values). The data showed a positive correlation with that of mRNA expression levels in both RF- and SH-exposed cells. In GR-exposed cells, the PARP-1 protein levels were significantly higher than in SH-exposed cells at 0 hours (i.e., ~10 minutes after exposure), 0.5 hours, and 1 hour but were similar and not significantly different between the two groups at 2, 4, 6, 8, and 10 hours after exposure.

## 4. Discussion

The poly(ADP-ribose) polymerase-1 (PARP-1) was the focus of research for numerous investigators since this nuclear enzyme has been shown to be involved in genomic instability, repair of DNA strand breaks, gene transcription, cell cycle progression, chromatin function, and cell death [3–11]. To the best of our knowledge, thus far, induction of PARP in cells exposed to nonionizing electromagnetic fields was not reported in the scientific literature. However, the results from two recent investigations have provided the evidence that a very low, nongenotoxic dose of IR was able to upregulate/increase the PARP-1 mRNA expression and its protein levels. The observations of Zhang et al. [13] included the following: (i) mice exposed to low dose IR (0.05 Gy ^12^C^6+^ ion beam) showed significantly increased PARP-1 enzyme activity and its protein levels while the incidence of chromosomal aberrations (CA) in spermatogonia and spermatocytes was similar to those in unexposed controls; (ii) mice exposed to high dose (2 Gy ^12^C^6+^ ion beam) showed significantly increased CA and decreased levels of PARP-1 activity and its protein; (iii) mice that received both low and high doses had significantly decreased CA and restored levels of PARP-1 activity and its protein; (iv) the effects observed in mice which received low and high doses were blocked when they were additionally injected with 3-AB (immediately after the low dose). Thus, the authors suggested that the increased PARP-1 activity and its protein might have played a role in decreasing the CA/genotoxicity in mice irradiated with low and high dose IR. In a more recent investigation, Cheng et al. [14] exposed cultured mouse lymphoma EL-4 cells to low (0.075 Gy) ± high doses (1, 1.5, and 2 Gy) of X-rays. Some cells were also treated with 3-AB one hour before low and high doses. The results indicated that the expression of PARP-1 and p53 mRNA and the protein levels as well as % cells in apoptosis were significantly increased in cells exposed to low dose compared with those exposed to high doses. In addition, treatment of the cells with 3-AB resulted in downregulation of PARP-1 and p53 and negated the effects induced by high dose X-rays. Thus, the authors concluded that PARP-1 and p53 may have played important roles in cells exposed to low dose X-rays.

The results obtained in our current study, indeed, suggested that nonionizing 900 MHz RF at 120 *μ*W/cm^2^ power intensity exposure for 3 hours/day for 5 days was capable of increasing/upregulating the PARP-1 mRNA expression and its protein levels in BMSCs (compared to SH- and GR-exposed cells), and their decreased levels from 0 hours to 10 hours may be due to their degradation over time. Such induction/degradation of PARP-1 mRNA expression and its protein was not observed in SH-exposed cells. In GR-exposed BMSCs, there was an increase in PARP-1 mRNA expression and protein levels at 0 hours, that is, ~10-minute intervening time between exposure and performing the assays. These increases may be due to the immediate response of the cells to the damage induced by GR exposure and/or due to the “stress” during the transport of cells from one building to the other. However, the subsequent decreases observed at 4 and 2 hours might be due to their use in the repair of GR-induced DNA strand breaks.

There were several reports indicating the involvement of PARP-1 in inducing apoptosis or programmed cell death [21–25] and PARP inhibitors are considered as potential therapeutic agents for life-threatening diseases [26, 27]. In the context of animal and human cells exposed to RF, there were contradictory reports on the induction of apoptosis [28–30]. In our earlier investigation, we did not observe induction of apoptosis when HL-60 cells were exposed to 900 MHz RF [19]. In the current investigation, the extent of apoptosis was not assessed, but this needs to be examined further in view of the increased PARP mRNA expression and PARP protein levels in BMSCs.

Exposure of animal and human cells to nonionizing RF may generate some “stress” which may cause undetectable DNA damage and may stimulate signal transduction pathways leading to the activation of cell defense mechanisms. The activated cell defenses provide the cells with the ability to resist higher level damage induced by subsequent exposure to genotoxic agents. Such defense, also referred to as adaptive response (AR), has been reported in animal and in human cells preexposed to RF (reviewed in [31, 32]). The data was from our most recent study in which mice which were preexposed to RF and then challenged with a genotoxic dose of BLM showed significantly reduced levels of strand breaks in the DNA as well as faster kinetics of their repair [15]. The increased PARP mRNA and PARP protein levels observed in the current study provide mechanistic evidence for such DNA damage repair and thus RF-induced AR. Nonetheless, this needs to be confirmed in appropriate RF-induced AR investigations, that is, animal and human cells preexposed to RF and then challenged with genotoxic agents.

## 5. Conclusion

The overall observations in our investigation indicated that nonionizing 900 MHz RF exposure at 120 *μ*W/cm^2^ power intensity in BMSCs was capable of increasing/upregulating the PARP-1 mRNA expression and its protein levels while such changes were not observed in SH-exposed cells.

## Figures and Tables

**Table 1:** Poly(ADP-ribose) polymerase-1 (PARP-1) mRNA expression levels, relative to housekeeping gene GADPH, in mouse bone marrow cells at different times following exposure to 900 MHz radiofrequency fields (RF), sham (SH), and 1.5 Gy gamma radiation (GR).

Times (h)	RF	SH	GR	*p* values		
				RF versus SH	RF versus GR	SH versus GR
0.0	11.26 ± 0.20	1.00 ± 0.00	2.78 ± 0.15	<0.05	<0.05	<0.05
0.5	10.42 ± 0.35	0.98 ± 0.01	2.33 ± 0.20	<0.05	<0.05	<0.05
1.0	9.13 ± 0.49	1.12 ± 0.10	2.18 ± 0.09	<0.05	<0.05	<0.05
2.0	8.68 ± 0.35	1.12 ± 0.09	1.79 ± 0.07	<0.05	<0.05	<0.05
4.0	7.94 ± 0.57	1.16 ± 0.10	1.72 ± 0.07	<0.05	<0.05	<0.05
6.0	7.71 ± 0.68	1.24 ± 0.15	1.63 ± 0.06	<0.05	<0.05	NS
8.0	7.64 ± 0.74	1.17 ± 0.13	1.56 ± 0.08	<0.05	<0.05	NS
10.0	7.40 ± 0.71	1.16 ± 0.10	1.45 ± 0.12	<0.05	<0.05	NS

Data are mean ± standard deviation from 3 separate experiments. Significant differences: *p* < 0.05; NS: not significant.

**Table 2:** Poly(ADP-ribose) polymerase-1 (PARP-1) protein, fold changes relative to housekeeping gene GADPH, in mouse bone marrow cells at different times following exposure to 900 MHz radiofrequency fields (RF), sham (SH), and 1.5 Gy gamma radiation (GR).

Times (h)	RF	SH	GR	*p* values		
				RF versus SH	RF versus GR	SH versus GR
0.0	3.55 ± 0.26	1.00 ± 0.00	1.97 ± 0.08	<0.05	<0.05	<0.05
0.5	4.26 ± 0.23	1.08 ± 0.08	1.87 ± 0.14	<0.05	<0.05	<0.05
1.0	3.82 ± 0.31	1.13 ± 0.05	1.40 ± 0.05	<0.05	<0.05	<0.05
2.0	3.50 ± 0.24	1.13 ± 0.04	1.08 ± 0.16	<0.05	<0.05	NS
4.0	3.27 ± 0.29	1.09 ± 0.05	1.05 ± 0.11	<0.05	<0.05	NS
6.0	2.54 ± 0.13	1.09 ± 0.01	1.09 ± 0.12	<0.05	<0.05	NS
8.0	2.42 ± 0.17	1.10 ± 0.02	1.01 ± 0.05	<0.05	<0.05	NS
10.0	2.30 ± 0.19	1.03 ± 0.07	1.05 ± 0.07	<0.05	<0.05	NS

Data are mean ± standard deviation from 3 separate experiments. Significant differences: *p* < 0.05; NS: not significant.
